# TOP TEN TIPS FROM THE EDITOR-IN-CHIEF OF AGEING & SOCIETY

**DOI:** 10.1093/geroni/igae098.0359

**Published:** 2024-12-31

**Authors:** Philip Taylor, Sandra Torres, Dialechti Tsimpida

**Affiliations:** University of Warwick, Coventry, England, United Kingdom; Uppsala University, Uppsala, Uppsala Lan, Sweden; University of Southampton, Southampton, England, United Kingdom

## Abstract

Understanding the nuances of academic writing genres is crucial to nailing down the craft that is writing for peer-review journal publication. In this presentation, we will leverage an Editor-in-Chief’s expertise, to explore the routines of esteemed international peer-review journals like Ageing & Society. By uncovering editors’ expectations, this session offers invaluable insights into mastering the art of writing for prestigious publications. More specifically, this presentation will shed light on different publication outlets, the different readers these cater to, and how these differences impact how we craft manuscripts about the research we conduct. Building upon this foundational understanding, we will delve into strategies for presenting data collected in specific national contexts, contributing to global discussions in social gerontology.The ultimate goal is to provide attendees with an editor-in-chief’s top 10 tips by focusing on the routines that established international peer-review journals, such as Ageing & Society utilize, and the expectations editors place on the different sections of a manuscript.

